# Development and implementation of an online community as a strategy for mixed methods research during a pandemic

**DOI:** 10.1186/s40900-022-00383-5

**Published:** 2022-09-05

**Authors:** Lisa Shea, Jennifer Bushen, Nina Ahmad, Gabrielle Geonnotti, Joy LaMori, Stephanie Terrey, Pepa Gonzalez, Jill Shuman

**Affiliations:** 1 Janssen Scientific Affairs, LLC, 800 Ridgeview Drive, Horsham, PA 19044 USA; 2 Janssen Scientific Affairs, LLC, Titusville, NJ USA; 3 CorEvitas, LLC, Waltham, MA USA

**Keywords:** Online health community, Qualitative research, Mixed methods, Pandemic, Vaccines

## Abstract

Conducting mixed methods research is critical for healthcare researchers to understand attitudes, behaviors, and experiences on health-related topics, such as vaccine acceptance. As the COVID-19 pandemic has made it difficult to employ traditional, face-to-face qualitative methodologies, this paper describes the use of a virtual platform to conduct person-centered research. To overcome these challenges and better understand the attitudes and behaviors of vaccine-eligible individuals in the United States, an online health community called the Virtual Engagement Research Community (VERC) was designed and implemented. Using the Health Belief Model as a framework, the VERC employed a mixed methods approach to elicit insights, which included discussion topics, rapid polls, and surveys. Throughout the initial enrollment period of April–October 2021, continuous improvement efforts were made to bolster recruitment and member engagement. This agile research strategy was successful in utilizing mixed methods to capture community sentiments regarding vaccines. While this community focused on vaccination, the methodology holds promise for other areas of health research such as obesity, HIV, mental health disorders, and diabetes.

## Plain English summary

The COVID-19 pandemic has made it difficult to conduct research in person, but qualitative and quantitative data remain critical for developing research design. To gather and understand vaccine perceptions, opinions, and behaviors in a real time manner, The Virtual Engagement Research Community (VERC) was launched. As of October 2021, the VERC had 72 members who had the opportunity to participate in research events such as surveys, polls, discussion topics, and moderated chats in a private, invitation-only virtual community. The online format offered researchers the opportunity to adapt the types and frequency of engagements, fine-tune the research questions based on community responses and current events, and adjust the analysis and reporting methodology as needed to increase participation and maximize the value of the VERC. The success of the VERC and the lessons learned support the use of online communities to enhance mixed methods research as a valuable strategy for other health researchers.

## Background

During a pandemic there are barriers to carrying out in-person research methodologies, especially in healthcare [1, 2]. Stakeholders (e.g., public health authorities, healthcare providers, research teams, and potential participants) may be preoccupied with providing care, dealing with resource constraints, or struggling with the personal impact of a pandemic. Furthermore, carrying out fieldwork during a pandemic could expose researchers or participants to infection, especially if close contact with affected communities or health care facilities occurs. The progression of the COVID-19 pandemic created a rapidly evolving landscape, which required research methodologies to quickly adapt to real-time needs. It challenged researchers to identify new ways to recruit and engage participants, for both qualitative and quantitative research [3]. The pandemic also revealed new research topics, such as how and why individuals are adopting safety behavior recommendations and mandates, attitudes towards vaccines in development, as well as what information sources are deemed credible. Lastly, given the heightened sensitivity in the environment adding to the complexity of the vaccine decision-making process, research to understand attitudes and behaviors towards the COVID-19 vaccine became critical [4].

## Main text

### Determining a solution

The US Medical Affairs vaccine team at Janssen, the pharmaceutical company of Johnson & Johnson, has a commitment to conduct person-centered research to better understand vaccine perceptions, opinions, and behaviors, and how these may change over time given the dynamic environment. To achieve this after the COVID-19 pandemic began, the team partnered with HealthiVibe, the patient experience unit of CorEvitas LLC, to employ a methodology to continue its commitment to patient engagement research. The following criteria were considered necessary for methodological success:

- Engagement with the same individuals over time
- Easily accessible Internet platform so participants can engage from any computer or digital device anywhere
- Demographic and geographic diversity among participants
- Agility to adapt research topics in a changing landscape
- Integration of both qualitative and quantitative research methods
- Interactivity of participants with researchers and other participants
- Anonymity of participants to engage with researchers and other participants openly

These elements are all characteristic of online health communities (OHCs), which offer a virtual environment to capture longitudinal insights from a diverse group of people. In these communities, individuals can be asked to participate anonymously and asynchronously in discussion topics and moderated chats, and to complete surveys and polls. These capabilities allow researchers to employ a mixed methods approach to generate a robust data set that other methodologies, such as telephone or video interviews, may not offer [5–8]. While numerous studies and health promotion efforts have been conducted to understand the factors associated with vaccine acceptance and/or hesitancy prior to the COVID-19 pandemic [9, 10], there is a paucity of data regarding utilizing OHCs to capture individuals’ attitudes, opinions, and experiences regarding vaccines. Therefore, the objective of our intervention was to understand the potential strengths and limitations of a private OHC as a nimble approach to generate evidence.

**Fig. 1:**
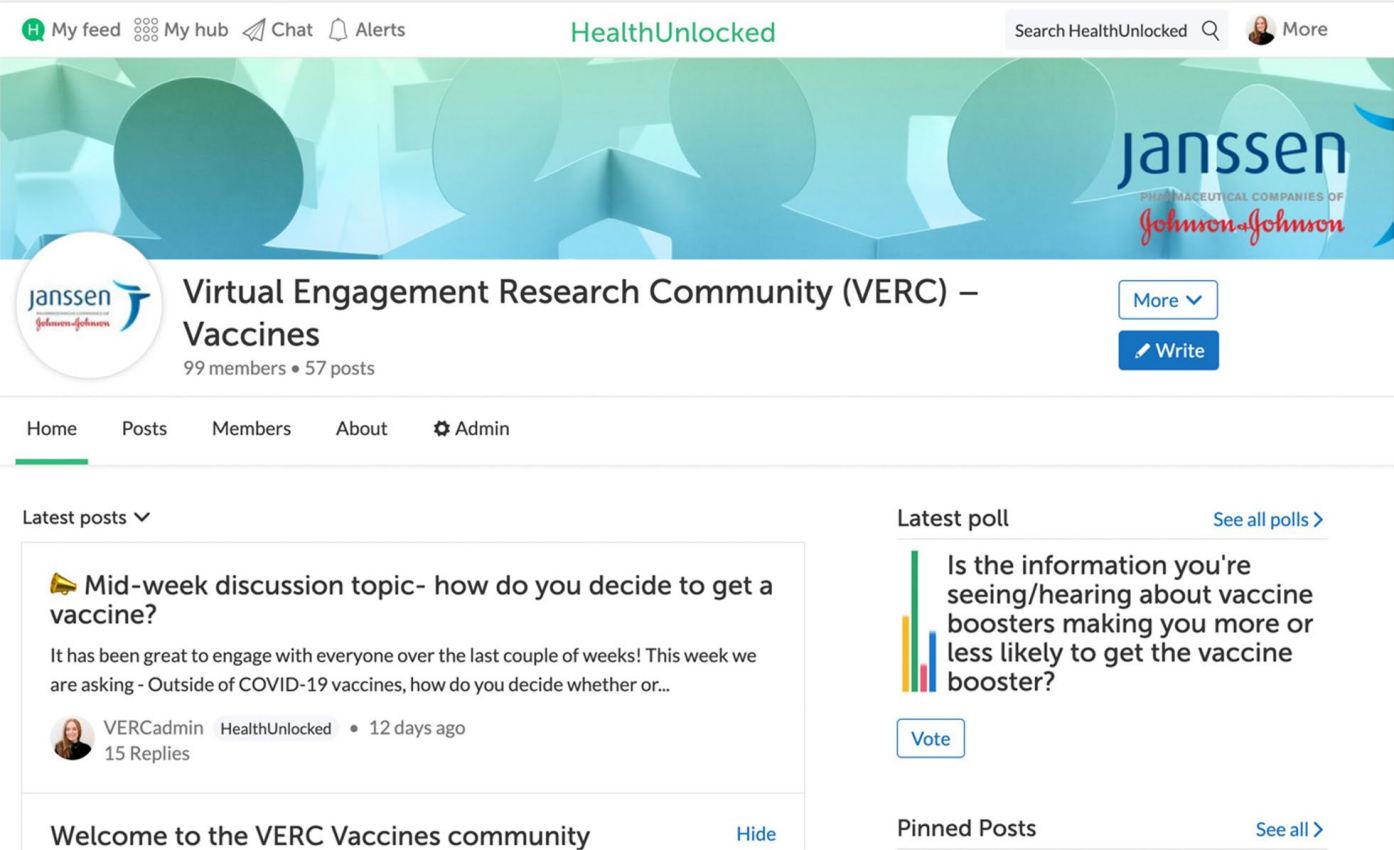
VERC community homepage

The OHC is called the “Virtual Engagement Research Community” (VERC) and is hosted on CorEvitas's social network for health, HealthUnlocked (HU; www.healthunlocked.com). HealthUnlocked is an accessible, established social network platform for health, comprising more than 1.5 million members and 315 public peer-to-peer support communities. The VERC is the only vaccine community within HealthUnlocked and is an invitation-only space. The homepage of the VERC (Fig. 1) has several features, including space to respond to discussion topics, vote in rapid polls, and search for previous posts via keywords. Chat functionality enables participants to have live, direct discussions with moderators.

### Developing and launching the online health community

Inclusion and exclusion criteria were developed based on the publicly available guidance for the COVID-19 vaccines provided by the Advisory Committee on Immunization Practices (ACIP) of the Centers for Disease Control and Prevention (CDC). Table 1 outlines the inclusion and exclusion criteria, and Table 2 shows the target and current demographic mix, which prioritized individuals across different race/ethnic groups, various age ranges, and employment status. A recruitment plan was developed as well as an online screener, which was sent to current members of the HealthUnlocked platform. Individuals who expressed interest in joining the VERC completed the screener and invitations to join the VERC were sent based on the screener responses against the criteria and target demographics. Recruitment began in April 2021 and by October 2021, there were 72 members enrolled and engaged in the VERC. Table 3 shows the recruitment funnel detailing the number of screeners completed, number of invitations sent, and members enrolled as of October 31, 2021.

**Table 1:** Inclusion and exclusion criteria

Inclusion criteria	Exclusion criteria
COVID-19 vaccine-eligible^a^ individuals aged 18 and older United States resident	Currently employed by a pharmaceutical company or regulatory agency or have an immediate family employed at one Currently serving on a vaccine advisory board or advisory committee Candidates who rate themselves low on measure of tech-savviness (scale of 1–5) Candidates who rank themselves as completely opposed to vaccinations (scale of 1–5) Individuals with cancer who are in active treatment

^a^Per the Advisory Committee on Immunization Practices (ACIP) of the Centers for Disease Control and Prevention (CDC). https://www.cdc.gov/vaccines/covid-19/clinical-considerations/covid-19-vaccines-us.html

**Table 2:** VERC recruitment targets versus community composition as of October 31, 2021

	Recruitment target N (%)	Community composition N (%)	US census percentage^a^
Race/ethnicity^b^			
White	45 (45)	46 (64)	75.8
Black	25 (25)	10 (14)	13.6
Latino/Hispanic	30 (30)	14 (19)	18.9
Asian	10 (10)	7 (10)	6.1
Employment type			
Healthcare provider (HCP)	20 (20)	13 (18)	N/A
Non-HCP essential worker^c^	20 (20)	10 (14)	N/A
Other employment	60 (60)	49 (68)	N/A
Health status			
Healthy	50 (50)	22 (31)	N/A
Underlying condition^d^	50 (50)	50 (69)	N/A

^a^https://www.census.gov/quickfacts/fact/table/US/PST045221

^b^Members could select more than one Race/Ethnicity

^c^Non-HCP essential workers include: Agriculture or food processing; Educator (example teacher, professor, early childhood education or childcare, etc.); Hospitality or restaurant; Retail Sales (example grocery store, clothing store, etc.); Tradesmen (construction, plumber, electrician, etc.); Transportation (bus driver, airline pilot, etc.); Police, corrections officer, firefighter, or other public safety

^d^Underlying conditions include: Cardiovascular Disease (For example but not limited to: Coronary Artery Disease, Heart Attack, Arrythmia, High Blood Pressure); Autoimmune Disease (For example but not limited to: Rheumatoid Arthritis, Lupus, Celiac Disease, Multiple Sclerosis, Ankylosing Spondylitis); Respiratory Disease (For example but not limited to: COPD, Chronic Bronchitis, Emphysema, Cystic Fibrosis); Cancer; Immunocompromising conditions; Diabetes; Overweight/obesity

**Table 3:** VERC recruitment funnel

Funnel	N
Screener responses	310
Invited to join VERC	183
All-time members	77
Current members	72

Participants electronically signed a consent document during the screening process. Because all data collected were de-identified, no ethics board review was required. Community guidelines were created and posted in the community with a disclaimer that the forum was not intended to discuss a specific company’s products or treatments, medical conditions, clinical trials, or financial results. HealthUnlocked’s Terms of Use and Privacy policy were readily available to members.

The VERC was designed to provide ongoing opportunities for community members to engage in research activities. Several different methodologies were used, which were categorized broadly as discussion topics and research events. Discussion topics were weekly qualitative questions posted on the VERC’s homepage. Members could reply to these questions as well comment on the replies of other members. The threads were moderated by research team members who would follow-up with questions and comments. Research events took place monthly and employed both qualitative and quantitative methodologies, including: rapid polls, surveys, live chats, and participant panels. The details of each engagement methodology can be seen in Table 4. The activities were intentionally scheduled to be more frequent at the start of the community to build engagement and then decrease slightly over time. Data were monitored on a weekly basis to capture the number of members who participated in discussion topics and research events.

**Table 4:** Description of research methodologies

	Discussion topics	Rapid polls	Surveys	Live chat	Participant panel
Description	Open-ended questions posed to entire community that members can respond to asynchronously	Single question survey posed to the entire community	10–15 question survey sent to all members’ email addresses	1–2 h synchronous discussion with invited members led by a HealthiVibe moderator	Week-long, asynchronous event where one discussion topic is posed daily to invited members in a dedicated page
Use	Generates discussion and builds community	Quick data generation	Standardized data generation	In-depth insights from a smaller group of members in real-time	In-depth insights from a smaller group of targeted members
Frequency	Weekly	Every other month	Every other month	Ad hoc	Ad hoc
Qualitative or quantitative	Qualitative	Quantitative	Quantitative	Qualitative	Qualitative

### Research content development

The VERC was conceptualized as a research platform to understand vaccine knowledge, attitudes, and behaviors. The initial research content was developed in early spring 2021, when the first COVID-19 vaccines were authorized for emergency use by the US Food and Drug Administration and vaccine recommendations were rolling out in phases throughout the United States. Therefore, initial priority topics were related to COVID-19 vaccine availability and willingness, vaccination experience, knowledge/information sources as well as general experiences during a novel pandemic (e.g., safety behaviors and mandates); topics later evolved to include boosters, variants, as well as impacts on healthcare and the workplace. Topics that garnered the most engagement typically were those that coincided with specific, real-time events. For example, discussion topics focused on potential boosters prompted the most responses from members as recommendations for these were being considered.

The development of the research content was grounded in the Health Belief Model (HBM), a widely accepted theoretical model that helps explain individuals’ willingness to adopt health interventions, including vaccines [11]. The key constructs within the HBM are perceived susceptibility, perceived severity, perceived benefits, barriers, self-efficacy, and cues to action. These are the beliefs an individual has that then drive their health behavior(s) [12–15]. Table 5 shows example of discussion topics and/or research events and the HBM construct they were designed to explore.

**Table 5:** Engagement questions by HBM construct

Construct	Engagement questions
Perceived susceptibility	When do you anticipate returning to your in-person location? How do you feel about returning in-person? (Discussion topic) Are you comfortable relaxing your social distancing and quarantine behaviors? What would make you feel more comfortable returning to "normal"? (Discussion topic)
Perceived severity	How concerned are you at what you're hearing about COVID-19 variants? What are your major concerns? (Discussion topic)
Perceived benefits	What questions do you have about COVID-19 vaccine boosters? What do you want to know? (Discussion topic) To what extent did each of the following reasons contribute to your decision to receive the COVID-19 vaccine? (Concern for personal health, health of family members, desire to help community, HCP recommendation, information from news/media, job/school requirements, pressure from family/friends) (Survey)
Perceived barriers	How difficult was it for you to schedule an appointment to receive the COVID-19 vaccine? (Discussion topic) What obstacles do you face in finding information on the COVID-19 vaccine? (Survey)
Perceived self-efficacy	Have you scheduled or received your COVID-19 vaccination? Why or why not? (Discussion topic) How knowledgeable do you consider yourself about the differences between the currently available vaccines? (Poll) If you have received the vaccine, did anyone help you to find a location or schedule your appointment? If so, who? (Discussion topic)
Cues to action	What have you been hearing about the COVID-19 vaccines lately? What would you like to hear that you haven’t? (Discussion topic) What factors could change your current decision on whether you will receive, not receive, or delay vaccination? (Live chat) What questions do you have about COVID-19 vaccine boosters? What do you want to know? (Discussion topic)

HBM, health belief model

Several strategies were employed to optimize the agility of the online community platform in response to the ever-changing environment. First, the community was moderated daily to ensure that emerging themes were identified. For example, research content was developed in response to member-generated discussion around COVID-19 boosters prior to formal recommendation from the CDC. Second, qualitative discussion topics were used to inform research events and vice versa. In one topic, several members shared their experiences with close family and friends who were vaccine hesitant. Those members were invited to a live chat and had an in-depth discussion that provided valuable insight into hesitancy within their communities. Finally, research content was adapted based on community members’ engagement patterns; some members responded to discussion topics, but not research events. Therefore, community members were asked about key topics in both formats to collect comprehensive quantitative and qualitative data.

## Discussion

Implementing any intervention requires careful planning, and OHCs are no exception. This OHC is not modeled directly on any previous research, but it was designed to build on prior experience with virtual research methods, focused on one diagnosis or health condition. No patients or members of the public were consulted as part of the design of this research or its analysis. This experience showed the importance for researchers to consider the circumstances of the population they are trying to reach. The VERC accomplished this by leveraging technology to gather insights into vaccine attitudes and behaviors at a time when other, more traditional methodologies would have proven difficult. Additionally, some key strengths of the OHC methodology allowed the researchers to gather these insights effectively, including:

- Allowing community members to engage anywhere and asynchronously, reducing logistical or geographic limitations and member burden
- Maintaining the anonymity of community members, which may increase their comfort sharing information and encourage honest participation, especially with politically charged and potentially sensitive topics
- Creating an agile and adaptive methodology that allows for learning throughout the process and implementation of continuous improvement
- Building diversity in types of engagements and topics, which can appeal to a broad audience and maintain interest
- Utilizing a mixed methods approach, which provides the advantages of both qualitative and quantitative research
- Granting members the opportunity to reply to other members’ comments and create their own posts, which provides opportunities for community building and organically emerging themes
- Providing ongoing engagement over a period of time, allowing the community to be leveraged for new topics (e.g. vaccines for other diseases) and creating opportunities to revisit topics for longitudinal comparison

There are, however, some important limitations to the OHC methodology to consider. For example:

- Lack of member compensation, relying instead on members’ interest in the topics and satisfaction/engagement with the platform
- Member comfort with technology and access to a computer and internet was necessary, which could introduce sample bias
- Communication is entirely written, which required members to have a higher degree of literacy
- The platform allows members to edit or wordsmith their comments before posting, which could allow them to alter their true response and make it more “socially acceptable”

The advantages to this person-centered research strategy outweigh the limitations; in particular, the agile platform allows for rapid response to the community sentiment and the ability to employ a mixed methods approach. Researchers interested in replicating this methodology should be prepared to evolve their strategies and thinking, but doing so allows the OHCs to be continually used throughout a longer period of time. While this paper focuses on the implementation of the VERC methodology and key lessons learned between April–October 2021, the platform is still active and research is ongoing.

## Lessons learned

The need for person-centered, qualitative and quantitative research was critical before the COVID-19 pandemic, but the circumstances since March 2020 have required nimble and evidence-based approaches [16, 17]. Developing and implementing an OHC during this time proved to be a successful method to better understand vaccine attitudes and behaviors. As the VERC progressed, three key lessons emerged from the methodology: recruitment, engagement, and data analysis/reporting.

The goal was to recruit 100 participants into a demographically diverse community and progress toward this goal was actively monitored. As of October 31, 2021, 72 members were enrolled in the community. The initial recruitment strategy focused on outreach to existing HealthUnlocked users who matched the eligibility criteria. This led to the community skewing towards mostly white individuals with underlying conditions who were greater than 50 years of age and with similar types of employment (e.g., healthcare providers or non-medical essential workers). To recruit younger members from different racial/ethnic and employment backgrounds, the HealthUnlocked team pursued outreach on other social media channels, sent personalized emails to racially/ethnically diverse people active in other HealthUnlocked communities, and leveraged strong relationships within the communities of interest. As a result, the number of racial/ethnic minority groups increased from less than 20% in April to 42% by October. It may, however, have resulted in sampling bias as members of HealthUnlocked may be more likely to be willing to discuss health related topics, limiting the generalizability of findings.

The VERC was designed to capture attitudes of those who were recommended a COVID-19 vaccine and may potentially choose to be vaccinated at the time of roll out. Therefore, certain exclusion criteria were decided upon. For example, potential members were excluded if they were actively being treated for cancer because there was no recommendation for immunocompromised populations at the time of enrollment. Other exclusion criteria were also applied. Members who reported themselves as ‘not-at-all’ or ‘somewhat’ comfortable with using technology were excluded from participating due to the online nature of the community and the different digital methods employed. Additionally, individuals who reported themselves as being ‘completely opposed to vaccination’ were excluded. It was hypothesized that excluding these potential members would allow the research team to more accurately assess the nuance of opposition to specific vaccines, such as the COVID-19 vaccine, and facilitate a continuous dialogue related to vaccines in general. Also this group would likely not be a group that would accept the COVID-19 vaccines during the roll out. While this resulted in a cohort that included individuals who were, at most, ‘somewhat opposed’ to vaccines, the initial results still indicate that the community members have a wide variety of attitudes towards vaccines. To correct for this potential bias, questions were asked related to vaccine opposition. For example, members were asked about opposition to vaccines among their families, social circles, and community. Opportunities may exist for future research to use OHCs to explore individuals’ vaccine opposition.

Community member engagement was initially strong throughout April and May, but began to wane in late June and July. It is not believed that this is due to drop out, as only 5 members had left the community between April and October 2021. This may have been multifactorial: posting weekly discussion topics may have been too frequent and/or members chose to log in less often; some members also may have felt uncomfortable commenting on politically charged topics or providing a minority opinion. Additionally, while beneficial to ask similar questions in both the discussion topics and the research events, community members may have felt this was redundant or that they had previously contributed similar information.

To re-engage members, several approaches were employed, including sending members email reminders and personal direct messages within HealthUnlocked, ‘tagging’ specific community members in discussion threads, increasing the length of time members could participate in research events, and conducting the research events that previously garnered more engagement (e.g., surveys). These adjustments successfully increased engagement on discussion topics and research events. Researchers interested in replicating this methodology could employ similar tactics to maintain engagement. While the VERC was able to be accessed through both computers and digital devices, data is not available to understand which platforms members used to access the community. Understanding how the platform is used is an opportunity for future research and this insight could help design more effective studies in the future.

Ongoing data analysis and reporting were performed to respond to the changing environment and data trends within the VERC. Initially, research events and discussion topics were reported on individually and at different cadences. Because community members’ contributions to the discussion topics were thoughtful and thorough, and members were responsive to moderators’ follow-up questions, it became clear that discussion topics and research events could inform and complement one another. For example, surveys were conducted to learn about trusted sources of information sources, while members provided more detailed insights about these sources in the discussion topics. Therefore, the analysis and reporting approach pivoted to focus more on emerging themes across all the data sources and compiled into a monthly report for review, discussion, and action. Researchers should consider analyzing all data sources regularly to generate robust and meaningful reporting to more effectively guide the development of research content.

## Conclusion

Online health communities such as the VERC appear to be effective for conducting vaccine-related research and may be a potential platform to conduct health research in other areas of public health. They provide an anonymous method to collect longitudinal insights from individuals who have conditions linked to an increased risk for stigmatization [18] including, but not limited to, obesity [19, 20], mental health disorders [21–23], HIV [24, 25], and both type 2 [19, 26] and juvenile diabetes [27, 28]. This is because the anonymous nature of OHCs may provide a’safe’ space for patients to share their experiences openly. Additionally, some conditions may make it harder for patients to engage in traditional qualitative work and this method allows researchers to pursue real-time research activities that could improve understanding of these patients’ experiences and help inform interventions designed to improve health outcomes among these populations. Further research is needed into the effectiveness of OHCs overall, which medical conditions or health behaviors could benefit most from this approach, and how to sustain and grow an engaged community over a longer period of time.

## Data Availability

Not applicable.

## Abbreviations

ACIP: Advisory Committee on Immunization Practices
CDC: Centers for Disease Control and Prevention
COPD: Chronic obstructive pulmonary disease
COVID-19: Coronavirus disease 2019
HBM: Health belief model
HCP: Healthcare provider
OHC: Online health community
VERC: Virtual Engagement Research Community
